# Data availability in articles published in *Epidemiologia e Serviços de Saúde*: a cross-sectional study, 2025

**DOI:** 10.1590/S2237-96222026v35e20263000.en

**Published:** 2026-05-13

**Authors:** Taís Freire Galvão, Marcus Tolentino Silva

**Affiliations:** 1 Universidade Estadual de Campinas, Faculdade de Ciências Farmacêuticas, Campinas, SP, Brazil Universidade Estadual de Campinas Universidade Estadual de Campinas Faculdade de Ciências Farmacêuticas Campinas SP Brazil; 2 Universidade de Brasília, Departamento de Saúde Coletiva, Brasília, DF, Brazil Universidade de Brasília Universidade de Brasília Departamento de Saúde Coletiva Brasília DF Brazil

**Keywords:** Scientific Integrity Review, Datasets as Topic, Periodicals as Topic, Peer Review, Cross-Sectional Studies., Revisión de Integridad Científica, Conjuntos de Datos como Asunto, Publicaciones Periódicas como Asunto, Revisión por Pares, Estudios Transversales.

## Abstract

**Objective:**

To assess the frequency and factors associated with data sharing in articles published in *Epidemiologia e Serviços de Saúde*.

**Methods:**

A cross-sectional study was conducted based on articles published in 2025 in the journal. Data were considered available when the statement reported deposit in a repository with persistent identifiers. Descriptive statistics for the variables and the odds ratio (OR) with 95% confidence intervals (95%CI) for data availability, according to variables with statistical significance, were calculated.

**Results:**

Of 124 articles published, data sharing occurred in 35 (28.2%; 95%CI 21.1; 36.7%). Most articles were submitted in 2024 (87.1%) and accepted in 2025 (76.6%), the year with greater data availability (p-value 0.017). The duration of peer review was longer in articles that shared data (median: 168 days; interquartile range: 130; 221 days) than in those without sharing (median: 135 days; interquartile range: 103; 172 days; p-value 0.025). Articles accepted in 2025 were more likely to share data (OR 4.40; 95%CI 1.41; 19.44; p-value 0.022), as were those with longer peer-review duration, in days (OR 1.01; 95%CI 1.00; 1.01; p-value 0.043). No factor remained associated with the outcome in the adjusted analysis.

**Conclusion:**

Over a quarter of the articles published in the journal shared their data, which was influenced by editorial factors. The requirement for a statement showed a limited effect on data sharing. Other strategies should be considered to increase adherence to open science in the journal.

## Resumen

**Objetivo:**

Analizar la frecuencia y los factores asociados al intercambio de datos en los artículos publicados en *Epidemiología y Servicios de Salud*.

**Métodos:**

Se realizó un estudio transversal a partir de artículos publicados en 2025 en la revista. Se consideraron disponibles los datos cuando la declaración indicaba un depósito en un repositorio con identificadores persistentes. Se calcularon estadísticas descriptivas de las variables y *odds ratio* (OR), con intervalos de confianza del 95% (IC95%), de la disponibilidad de los datos según las variables con significancia estadística.

**Resultados:**

De 124 artículos publicados, se compartieron datos en 35 (28,2%; IC95 % 21,1; 36,7%). La mayoría de los artículos fue sometida en 2024 (87,1%) y aceptada en 2025 (76,6%), año en que hubo mayor disponibilidad de datos (p-valor 0,017). La duración de la revisión por pares fue mayor en los artículos que compartieron datos (mediana: 168 días; rango intercuartílico: 130; 221 días) que en aquellos sin compartición (mediana: 135 días; rango intercuartílico: 103; 172 días; p-valor 0,025). Los artículos aceptados en 2025 tuvieron mayor probabilidad de compartir datos (OR 4,40; IC95% 1,41; 19,44; p-valor 0,022), así como aquellos con mayor duración de la revisión por pares, en días (OR 1,01; IC95% 1,00; 1,01; p-valor 0,043). Ningún factor se asoció con el desenlace en el análisis ajustado.

**Conclusión:**

Poco más de un cuarto de los artículos publicados en la revista compartió sus datos, lo cual estuvo influido por factores editoriales. La exigencia de declaración tuvo un efecto limitado en el intercambio de datos. Se deben considerar otras estrategias para aumentar la adhesión a la ciencia abierta en la revista.

## Introduction

Research data sharing is one of the components of open science that aims to enhance transparency and reproducibility. Various factors influence the availability of data used or generated in research, including culture and infrastructure maturity ^1^. Academic recognition of scientists committed to open science, at the expense of incentives for numerical productivity, appears to favor greater transparency ^2^. Developing a collaborative open science ecosystem requires commitment and proactivity from journals and other key stakeholders, such as funding agencies and research institutions ^3^.

The requirement for sharing data from clinical trials published in journals affiliated with the International Committee of Medical Journal Editors (ICMJE), introduced in 2016, has fostered this practice in the health field, albeit with variable adherence ^4^. Almost 70% of articles published between 2018 and 2020 in three prominent medical journals included a data availability statement, but less than 1% had anonymized individual patient data in publicly available repositories ^5^. Conditional access to data was frequently reported in articles published in other health journals, but fewer than two-thirds of corresponding authors confirmed willingness to share such data ^6^.

The discrepancy between stated intentions and actual data sharing-when access to data depends on direct requests to the authors-has proven to be of limited effectiveness across different disciplines ^7^. Most data requests conditioned on author contact were unsuccessful, with fewer than half to ^8^ one-third of messages receiving responses. ^9^. In the Brazilian setting, obtaining data for a systematic review on the prevalence of childhood obesity was successful in 31% of the 164 articles whose authors were contacted, requiring 3 months of contact before access was granted, on average, or the effort was abandoned ^10^.

In the second half of 2024, *Epidemiologia e Serviços de Saúde: revista do SUS* (RESS) updated its instructions for authors to include a data availability statement in published articles ^11^. Analyzing adherence to this practice would provide evidence to support the debate on open science in Brazil and empirical data to inform the editorial policies of this journal, which is one of the leading public health journals in the country.

This study aimed to assess the frequency and factors associated with data sharing in articles published in RESS in 2025.

## Methods

### Setting and participants

This is a cross-sectional study based on articles published in 2025 in RESS, belonging to volume 34, in the modalities of original article, research note, and review. 

### Variables

The study outcome, data availability, was considered present when the statement reported deposit in a recognized repository with a persistent identifier, such as the Digital Object Identifier (DOI) ^12^. The independent variables included editorial and methodological characteristics of the manuscripts: year of submission and acceptance (2024, 2025), duration of peer review (in months: <4, 4-6, >6), region (North, Northeast, Southeast, South, Central-West), interstate collaboration (yes, no), number of authors (2-4, 5-7, ≥8), female authorship (absent, minority, parity, majority), affiliation to health services (yes, no), main topic (COVID-19 and vaccination, nutrition, oral health, child health, other), and study design (cohort study, descriptive, cross-sectional, other).

### Data sources and measurement

Eligible articles were identified in the Medical Literature Analysis and Retrieval System Online (MEDLINE), via PubMed, using the search strategy “Epidemiol Serv Saude[jour] AND 2025[pdat]”, in January 2026. Based on the obtained DOI, web scraping of information for each article was performed from the Scientific Electronic Library Online (SciELO) using Python version 3.12.13 in the Google Colab environment. 

Authors’ affiliations supported the assignment of location and institutional affiliation. The state of the first author was considered to classify the region. Interstate publication was assigned when authors were from different states. Proportions of ≤33% female authors were considered minority; >33% and ≤66%, parity; and ≥66%, majority. Thematic classification was performed automatically using the hierarchical density-based spatial clustering of applications with noise (HDBSCAN) algorithm, which grouped keywords by similarity.

### Statistical methods

Categorical variables were described by absolute and relative frequencies, with comparisons between groups performed using Pearson’s chi-square test or Fisher’s exact test. The duration of peer review was analyzed as a continuous variable (in days) in inferential analyses, and its normality guided its description using the median and interquartile range; comparisons were performed using the Wilcoxon test.

The odds ratio (OR) and 95% confidence intervals (95%CI) for data availability for variables with statistical significance (p-value<0.05) in the descriptive analysis were calculated using logistic regression. Significant associations were included in the adjusted model to estimate the probabilities and 95%CI for data sharing, which were presented graphically. Missing data were left unaltered, and no sensitivity analyses were conducted. Python version 3.14.2 was used for variable extraction, measurement, and categorization, and R version 4.5.2 for data analysis.

## Results

Of the 172 articles published in RESS in 2025, 134 appeared in volume 34, and 124 were eligible and included in the analysis (Figure 1). Data sharing occurred in 35 articles (28.2%; 95%CI 21.1%; 36.7%).

**Figure 1:**
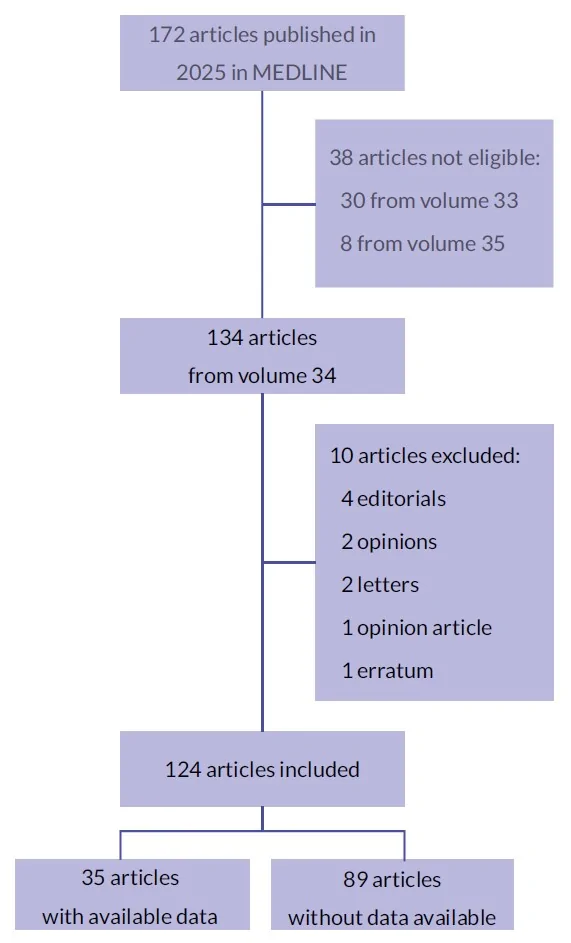
Process of identification and inclusion of articles in the study

Most articles were submitted in 2024 (87.1%) and accepted in 2025 (76.6%), the year with greater data availability (p-value 0.017). Peer review duration between four and six months (36.3%), submissions from the Southeast region (38.7%), from institutions based in a single state (65.3%), with five to seven authors (42.7%), with gender parity (53.2%), and cross-sectional design (47.6%) were more frequent. Slightly less than half of the articles had authors affiliated with health services (46.0%) (Table 1).

The duration of peer review was longer in studies that shared data (median: 168 days; interquartile range: 130; 221 days) than in those that did not (median: 135 days; interquartile range: 103; 172 days; p-value 0.025). Normality was observed in the distribution of the first group (p-value 0.930) and a non-normal distribution in the second (p-value 0.003).

**Table 1:** Characteristics of the included articles and frequency of data availability

Variable	Total	Available data	p-value
	n (%)	n (%)	
**Year of submission**			0.238^a^
2024	108 (87.1)	28 (80.0)	
2025	16 (12.9)	7 (20.0)	
**Year of acceptance**			0.017^b^
2024	29 (23.4)	3 (8.6)	
2025	95 (76.6)	32 (91.4)	
**Duration of peer review (months)**			0.062^a^
<4	42 (33.9)	7 (20.0)	
4-6	45 (36.3)	13 (37.1)	
>6	37 (29.8)	15 (42.9)	
**Region**			0.655^b^
North	2 (1.6)	1 (2.9)	
Northeast	35 (28.3)	12 (34.3)	
Southeast	48 (38.7)	11 (31.4)	
South	20 (16.1)	5 (14.3)	
Central-West	19 (15.3)	6 (17.1)	
**Interstate**			0.127^a^
Yes	43 (34.7)	8 (22.9)	
No	81 (65.3)	27 (77.1)	
**Number of authors**			0.371^a^
2-4	43 (34.7)	14 (40.0)	
5-7	53 (42.7)	16 (45.7)	
≥8	28 (22.6)	5 (14.3)	
**Female authorship**			0.349^a^
Absent	10 (8.1)	5 (14.3)	
Minority	21 (16.9)	7 (20.0)	
Parity	66 (53.2)	17 (48.6)	
Majority	27 (21.8)	6 (17.1)	
**Affiliation to health services**			0.814^a^
Yes	57 (46.0)	15 (42.9)	
No	67 (54.0)	20 (57.1)	
**Topics**			0.233^b^
COVID-19 and vaccination	11 (8.9)	3 (8.6)	
Nutrition	28 (22.6)	4 (11.4)	
Oral health	7 (5.6)	1 (2.9)	
Child health	14 (11.3)	6 (17.1)	
Other	64 (51.6)	21 (60.0)	
**Study design**			0.776^b^
Cohort	12 (9.7)	2 (5.7)	
Descriptive	16 (12.9)	5 (14.3)	
Cross-sectional	59 (47.6)	16 (45.7)	
Other	37 (29.8)	12 (34.3)	
**Total**	124 (100.0)	35 (100.0)	

a Pearson’s chi-square test; ^b^Fisher’s exact test.

Articles accepted in 2025 were more likely to share data (OR 4.40; 95%CI 1.41; 19.44; p-value 0.022), as were those with longer peer-review duration, in days (OR 1.01; 95%CI 1.00; 1.01; p-value 0.043). In the model adjusted for these variables, no statistically significant association was observed.

The probability of data sharing was higher in 2025 and increased with peer review duration, despite overlapping confidence intervals (Figure 2).

**Figure 2:**
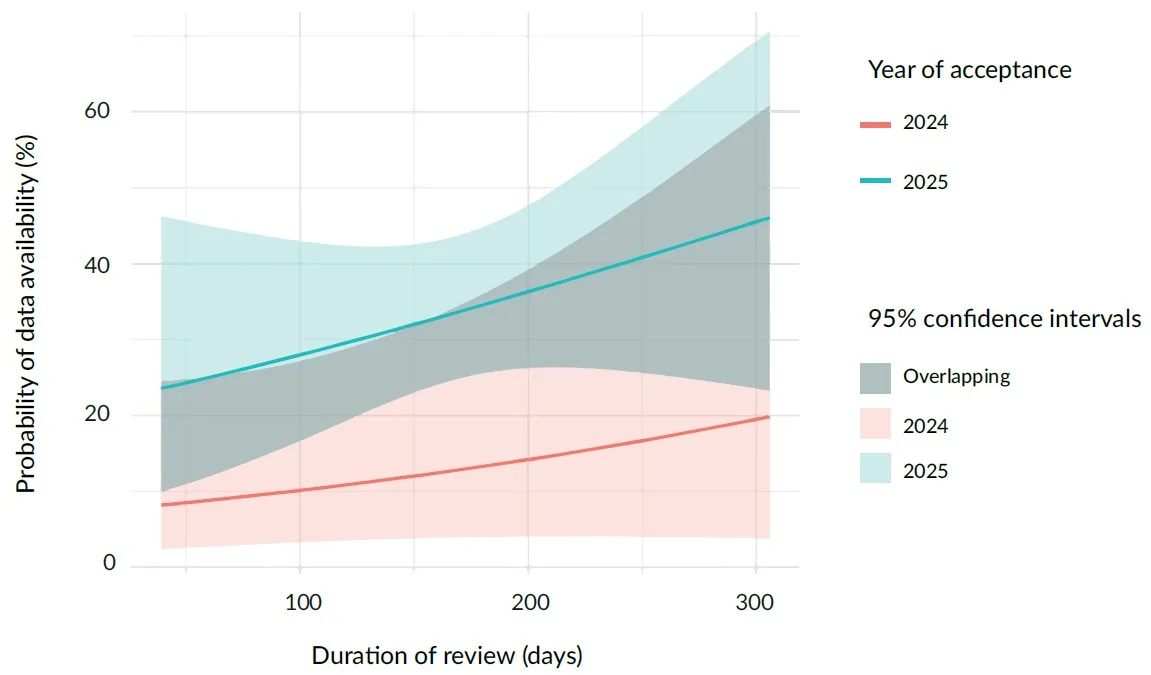
Probability of data availability by acceptance year (n=124)

## Discussion

Slightly over a quarter of the articles published in RESS in 2025 shared the data generated or used in the research. Articles accepted in 2025 and with longer processing times were more likely to adhere to this open science practice. These factors were not associated after adjustment. Other editorial or article characteristics did not influence data availability.

The primary outcome was based on the presence of persistent identifiers, such as a DOI, in the “Data Availability” section of articles published in RESS, without verification of content or assessment of data quality. In previous analyses, a simple data availability statement did not guarantee access to the underlying data in journals affiliated to ICMJE ^5^. The restriction to a single journal and the small sample size also limit how broadly the findings can be applied and the robustness of statistical analyses, which did not remain significant after adjustment.

The presence of a data availability statement in articles published in health journals was similar to that found in this analysis, with greater adherence observed over time ^6^^,^^7^^,^^9^. Across studies, data availability statements were significantly more frequent in journals with mandatory policies ^8^^,^^13^, and these articles were more likely to make research data available in repositories ^13^. 

A higher proportion of data sharing was observed in more recently accepted articles, possibly related to changes in editorial guidelines or greater adherence by authors to journal requirements ^11^. This result reinforces the role of mandatory editorial policies and institutional support mechanisms in promoting data sharing ^8^^,^^9^^,^^13^.

Each additional day of peer review increased the likelihood of data sharing by 1%. This effect, besides being marginal, was not associated with data sharing in the adjusted model, indicating that review time alone does not explain adherence to this good research practice. The absence of associations in the multivariate model may reflect sample size limitations, as well as unexamined factors such as technical limitations, time and resource constraints, ethical concerns, and fear of misuse of information ^1^^,^^9^. 

In conclusion, data sharing in RESS articles was limited, with data accessible in a quarter of 2025 studies. The requirement for a statement showed a limited effect on data availability. More effective approaches, such as requiring data deposition, should be implemented to enhance compliance with open science practices in the journal.

## Data Availability

The entire dataset supporting the results of this study is available on the Open Science Framework at https://doi.org/10.17605/OSF.IO/2YB6W.
