# Supplementary material for: Data availability in articles published in Epidemiologia e Serviços de Saúde: a cross-sectional study, 2025
Source: Epidemiol Serv Saude. 2026 May 13;35:e20263000. doi: 10.1590/S2237-96222026v35e20263000.en (PMC13686419; doi:10.1590/S2237-96222026v35e20263000.en)
Supplement: Translation

# Translation: Disponibilidade de dados em artigos publicados na ***Epidemiologia e Serviços de Saúde***: estudo transversal, 2025

**DOI:** 10.1590/S2237-96222026v35e20263000.pt

**Authors:** Taís Freire Galvão, Marcus Tolentino Silva

**Affiliations:** 1 Universidade Estadual de Campinas, Faculdade de Ciências Farmacêuticas, Campinas, SP, Brasil; 2 Universidade de Brasília, Departamento de Saúde Coletiva, Brasília, DF, Brasil

**Keywords:** Revisão de Integridade Científica, Conjuntos de Dados como Assunto, Publicações Periódicas como Assunto, Revisão por Pares, Estudos Transversais.

## Abstract

**Objetivo:**

Analisar a frequência e fatores associados ao compartilhamento de dados em artigos publicados na *Epidemiologia e Serviços de Saúde*.

**Métodos:**

Realizou-se estudo transversal a partir de artigos publicados em 2025 no periódico. Dados foram considerados disponíveis quando a declaração informava depósito em repositório com identificadores persistentes. Calcularam-se a estatística descritiva das variáveis e a razão de chances (*odds ratio*, OR), com intervalos de confiança de 95% (IC95%), da disponibilidade dos dados pelas variáveis com significância estatística.

**Resultados:**

De 124 artigos publicados, houve compartilhamento de dados em 35 (28,2%; IC95% 21,1; 36,7%). A maioria dos artigos foi submetida em 2024 (87,1%) e aceita em 2025 (76,6%), ano em que houve maior disponibilidade de dados (p-valor 0,017). A duração da revisão por pares foi maior em artigos que compartilharam os dados (mediana: 168 dias; intervalo interquartil: 130; 221 dias) do que naqueles sem compartilhamento (mediana: 135 dias; intervalo interquartil: 103;172 dias; p-valor 0,025). Artigos aceitos em 2025 tiveram maior chance de compartilhamento de dados (OR 4,40; IC95% 1,41; 19,44; p-valor 0,022), assim como aqueles com maior duração da revisão por pares, em dias (OR 1,01; IC95% 1,00; 1,01; p-valor 0,043). Nenhum fator se manteve associado ao desfecho na análise ajustada.

**Conclusão:**

Pouco mais de um quarto dos artigos publicados na revista compartilhou seus dados, o que foi influenciado por fatores editoriais. A exigência de declaração mostrou efeito limitado no compartilhamento de dados. Outras estratégias devem ser consideradas para aumentar a adesão à ciência aberta na revista.

## Introdução

O compartilhamento de dados de pesquisas é um dos componentes da ciência aberta que visa maior transparência e reprodutibilidade. Diferentes fatores influenciam na disponibilidade dos dados utilizados ou gerados em uma pesquisa, incluindo cultura e maturidade da infraestrutura ^1^. O reconhecimento acadêmico de cientistas que se comprometem com a ciência aberta, em detrimento do incentivo à produtividade numérica, parece favorecer maior transparência ^2^. Desenvolver um ecossistema colaborativo de ciência aberta requer compromisso e proatividade de periódicos e outros atores importantes, como agências de fomento e instituições de pesquisa ^3^.

A exigência de compartilhamento de dados gerados por ensaios clínicos publicados nos periódicos membros do International Committee of Medical Journal Editors (ICMJE) em 2016 impulsionou essa prática na área da saúde, com adesão variável ^4^. Quase 70% dos artigos publicados entre 2018 e 2020, em três proeminentes periódicos médicos, apresentavam declaração de disponibilidade de dados, mas menos de 1% deles tinha dados individuais de pacientes anonimizados e em repositórios de acesso público ^5^. O acesso condicional aos dados foi frequentemente informado em artigos publicados em outras revistas da área de saúde, mas a disposição em compartilhar tais dados foi confirmada por menos de dois terços dos autores correspondentes ^6^.

A divergência entre a declaração de intenção e o compartilhamento efetivo - situação em que o acesso aos dados depende de solicitação direta aos autores - revela-se pouco efetiva em diferentes disciplinas ^7^. A maioria das solicitações de dados condicionada a contato com autores mostrou-se infrutífera, com menos da metade ^8^ a um terço de respostas às mensagens ^9^. No contexto nacional, a obtenção de dados para uma revisão sistemática sobre prevalência de obesidade infantil foi bem-sucedida em 31% de 164 artigos cujos autores foram contatados, demandando em média três meses de contato até o acesso ou desistência ^10^.

No segundo semestre de 2024, a *Epidemiologia e Serviços de Saúde: revista do SUS* (RESS) atualizou suas instruções aos autores, incluindo declaração de disponibilidade de dados nos artigos publicados ^11^. Analisar a adesão a essa prática forneceria evidências para embasar o debate sobre ciência aberta no Brasil, bem como dados empíricos para retroalimentar as políticas editoriais desse periódico, que é um dos principais da área de saúde coletiva do país.

Esta pesquisa objetivou analisar a frequência e fatores associados ao compartilhamento de dados em artigos publicados na RESS em 2025.

## Métodos

### Delineamento e participantes

Trata-se de estudo transversal a partir de artigos publicados em 2025 na RESS, pertencentes ao volume 34, nas modalidades artigo original, nota de pesquisa e revisão. 

### Variáveis

Dados disponíveis, desfecho do estudo, foram considerados presentes quando a declaração informava depósito em repositório reconhecido e com identificadores persistentes, como o Digital Object Identifier (DOI) ^12^. As variáveis independentes incluíram características editoriais e metodológicas dos manuscritos: ano de recebimento e aceite (2024, 2025), duração da revisão por pares (em meses: <4, 4-6, >6), região (Norte, Nordeste, Sudeste, Sul, Centro-Oeste), interestadual (sim, não), número de autores (2-4, 5-7, ≥8), autoria feminina (ausente, minoria, paridade, maioria), vínculo com serviços de saúde (sim, não), tema principal (covid-19 e vacinação, nutrição, saúde bucal, saúde infantil, outras) e delineamento (estudo de coorte, descritivo, transversal, outros).

### Fontes de dados e mensuração

Os artigos elegíveis foram identificados na base Medical Literature Analysis and Retrieval System Online (MEDLINE), via PubMed, mediante estratégia de busca “Epidemiol Serv Saude[jour] AND 2025[pdat]”, em janeiro de 2026. A partir do DOI obtido, realizou-se raspagem de dados (*web scraping*) das informações correspondentes a cada artigo na Scientific Electronic Library Online (SciELO), por meio de linguagem Python, versão 3.12.13, em ambiente Google Colab. 

As afiliações dos autores embasaram a atribuição de localidade e vínculo institucional. O estado do primeiro autor foi considerado para classificar a região. Publicação interestadual foi atribuída para autoria de diferentes estados. Proporções de ≤33% de autoras do sexo feminino foram consideradas minoria; entre >33 e ≤66%, paridade; e ≥66%, maioria. A classificação temática foi realizada de forma automatizada, com o uso do algoritmo agrupamento espacial baseado em densidade com aplicações para ruído, de forma hierárquica (*hierarchical density-based spatial clustering of applications with noise*, HDBSCAN), que agrupou as palavras-chave por similaridade.

### Métodos estatísticos 

As variáveis categóricas foram descritas por frequências absolutas e relativas, com comparações entre grupos realizadas por meio do teste do qui-quadrado de Pearson ou do teste exato de Fisher. A duração da revisão por pares foi analisada como variável contínua, em dias, nas análises inferenciais, e a normalidade dessa variável orientou a descrição por mediana e intervalo interquartil, com comparações por meio do teste de Wilcoxon.

Foram calculados a razão de chances (*odds ratio*, OR) e intervalos de confiança de 95% (IC95%) da disponibilidade dos dados pelas variáveis com significância estatística (p-valor<0,05) na análise descritiva, por meio de regressão logística. As associações significativas compuseram modelo ajustado para estimar as probabilidades e o IC95% do compartilhamento de dados, tendo sido apresentadas graficamente. Dados ausentes não sofreram imputações e não houve análise de sensibilidade. Empregou-se o Python versão 3.14.2 para extração, mensuração e categorização das variáveis, e o R versão 4.5.2 para análise dos dados.

## Resultados

De 172 artigos publicados na RESS em 2025, 134 pertenciam ao volume 34, e 124 foram elegíveis e incluídos na análise (Figura 1). Houve compartilhamento de dados em 35 artigos (28,2%; IC95% 21,1%; 36,7%).

**Figura 1:** Processo de identificação e inclusão dos artigos no estudo

A maioria dos artigos foi submetida em 2024 (87,1%) e aceita em 2025 (76,6%), ano em que houve maior disponibilidade dos dados (p-valor 0,017). Foram mais frequentes tempo de revisão por pares entre quatro e seis meses (36,3%), submissões provenientes da região Sudeste (38,7%), de instituições sediadas em um único estado (65,3%), com cinco a sete autores (42,7%), com paridade de sexo (53,2%) e delineamento transversal (47,6%). Pouco menos da metade dos artigos tinham autores de serviços de saúde (46,0%) (Tabela 1).

A duração da revisão por pares foi maior em estudos que compartilharam os dados (mediana: 168 dias; intervalo interquartil: 130; 221 dias) do que naqueles sem compartilhamento (mediana: 135 dias; intervalo interquartil:103;172 dias; p-valor 0,025). Observou-se normalidade na distribuição do primeiro grupo (p-valor 0,930) e distribuição não normal no segundo (p-valor 0,003).

**Tabela 1:** Características dos artigos incluídos e frequência de disponibilidade de dados

Variável	Total	Dados disponíveis	p-valor
	n (%)	n (%)	
**Ano de recebimento**			0,238^a^
2024	108 (87,1)	28 (80,0)	
2025	16 (12,9)	7 (20,0)	
**Ano de aceite**			0,017^b^
2024	29 (23,4)	3 (8,6)	
2025	95 (76,6)	32 (91,4)	
**Duração da revisão por pares (meses)**			0,062^a^
<4	42 (33,9)	7 (20,0)	
4-6	45 (36,3)	13 (37,1)	
>6	37 (29,8)	15 (42,9)	
**Região**			0,655^b^
Norte	2 (1,6)	1 (2,9)	
Nordeste	35 (28,3)	12 (34,3)	
Sudeste	48 (38,7)	11 (31,4)	
Sul	20 (16,1)	5 (14,3)	
Centro-Oeste	19 (15,3)	6 (17,1)	
**Interestadual**			0,127^a^
Sim	43 (34,7)	8 (22,9)	
Não	81 (65,3)	27 (77,1)	
**Número de autores**			0,371^a^
2-4	43 (34,7)	14 (40,0)	
5-7	53 (42,7)	16 (45,7)	
≥8	28 (22,6)	5 (14,3)	
**Autoria feminina**			0,349^a^
Ausente	10 (8,1)	5 (14,3)	
Minoria	21 (16,9)	7 (20,0)	
Paridade	66 (53,2)	17 (48,6)	
Maioria	27 (21,8)	6 (17,1)	
**Vínculo com serviço de saúde**			0,814^a^
Sim	57 (46,0)	15 (42,9)	
Não	67 (54,0)	20 (57,1)	
**Temas**			0,233^b^
Covid-19 e vacinação	11 (8,9)	3 (8,6)	
Nutrição	28 (22,6)	4 (11,4)	
Saúde bucal	7 (5,6)	1 (2,9)	
Saúde infantil	14 (11,3)	6 (17,1)	
Outros	64 (51,6)	21 (60,0)	
**Delineamento**			0,776^b^
Coorte	12 (9,7)	2 (5,7)	
Descritivo	16 (12,9)	5 (14,3)	
Transversal	59 (47,6)	16 (45,7)	
Outros	37 (29,8)	12 (34,3)	
**Total**	124 (100,0)	35 (100,0)	

a Teste do qui-quadrado de Pearson; ^b^Teste exato de Fisher.

Artigos aceitos em 2025 tiveram maior chance de compartilhamento de dados (OR 4,40; IC95% 1,41; 19,44; p-valor 0,022), assim como aqueles com maior duração da revisão por pares, em dias (OR 1,01; IC95% 1,00; 1,01; p-valor 0,043). No modelo ajustado por essas variáveis, não houve associação estatisticamente significativa.

A probabilidade de compartilhamento de dados foi maior em 2025, aumentando com o tempo de revisão por pares, apesar da sobreposição dos intervalos de confiança (Figura 2).

**Figura 2:** Probabilidade de disponibilidade de dados pelo ano de aceite (n=124)

## Discussão

Pouco mais de um quarto dos artigos publicados na RESS em 2025 compartilhou os dados gerados ou utilizados na pesquisa. Artigos aceitos em 2025 e com maior tempo de processamento tiveram maior adesão a essa prática de ciência aberta. Tais fatores não se mantiveram associados após ajustes. Demais variáveis editoriais ou de características dos artigos não influenciaram na disponibilização dos dados.

O desfecho principal baseou-se na presença de identificadores persistentes, como DOI, na seção “Disponibilidade de dados” dos artigos publicados na RESS, sem conferência do conteúdo ou julgamento de qualidade dos dados. Em análises prévias, a simples declaração de disponibilidade não assegurou o acesso aos dados subjacentes em periódicos membros do ICMJE ^5^. A restrição a um único periódico e o pequeno tamanho amostral também limitam a generalização dos achados e a robustez das análises estatísticas, que não mantiveram significância após ajustes.

A presença de declaração de disponibilidade de dados, em artigos publicados em periódicos da área da saúde, foi semelhante à encontrada nesta análise, sendo observada maior adesão com o passar do tempo ^6^^,^^7^^,^^9^. Em diferentes análises, declarações de disponibilidade de dados foram significativamente maiores em periódicos com políticas mandatórias ^8^^,^^13^, e esses artigos apresentaram maiores chances de disponibilizar os dados de pesquisa em repositórios ^13^. 

Observou-se maior proporção de compartilhamento em artigos aceitos mais recentemente, possivelmente relacionada a mudanças nas orientações editoriais ou à maior aderência dos autores às exigências da revista ^11^. Esse resultado reforça o papel de políticas editoriais mandatórias e de mecanismos institucionais de suporte na promoção do compartilhamento de dados ^8^^,^^9^^,^^13^.

Cada dia adicional de revisão por pares aumentou em 1% a chance de compartilhamento de dados. Esse resultado, além de marginal, não se manteve associado ao compartilhamento de dados no modelo ajustado, indicando que o tempo de revisão, isoladamente, não explica a adesão a essa boa prática em pesquisa. A ausência de associações no modelo multivariado sugere a influência do tamanho da amostra, bem como de fatores não analisados, como limitações técnicas, restrições de tempo e recursos, preocupações éticas e receio de uso indevido das informações ^1^^,^^9^. 

Em conclusão, o compartilhamento de dados dos artigos publicados na RESS mostrou-se limitado, acessível em um quarto dos estudos publicados em 2025. A obrigatoriedade de declaração revelou-se limitada na disponibilização de dados. Estratégias mais eficientes, como a exigência do depósito de dados, devem ser consideradas para aumentar a adesão à ciência aberta no periódico.

## Data Availability

Todo o conjunto de dados que dá suporte aos resultados deste estudo foi disponibilizado no Open Science Framework e pode ser acessado em: https://doi.org/10.17605/OSF.IO/2YB6W.
